# Supplementary material for: Living with bronchial asthma: A qualitative study among patients in a hill village in Nepal
Source: PLoS One. 2023 Oct 20;18(10):e0291265. doi: 10.1371/journal.pone.0291265 (PMC10588826; doi:10.1371/journal.pone.0291265)
Supplement: S1 Appendix — (DOCX) [file pone.0291265.s002.docx]

Kindly introduce yourself

How long have you had bronchial asthma? According to you has it had an impact on your life?

How many attacks do you get in a year? When do you have more attacks?

Has asthma affected your ability to carry out your tasks?

Has it impacted your social life? Are you fully involved in village activities?

What do you know about the disease?

How do you obtain treatment? Do you face any difficulties doing so? Do you have to purchase your medicines?

Do you face financial problems because of your disease?

Any other issues you would like to discuss?

कृपया आफ्नो परिचय दिनुहोस्

तपाईलाई दम भएको कति भयो? तपाईका अनुसार यसले तपाईको जीवनमा प्रभाव पारेको छ ?

तपाइँ एक वर्षमा कति आक्रमणहरू प्राप्त गर्नुहुन्छ? तपाईलाई कहिले बढी आक्रमण हुन्छ?

के दमले तपाईंको कार्यहरू पूरा गर्ने क्षमतालाई असर गरेको छ?

के यसले तपाईको सामाजिक जीवनलाई असर गरेको छ? के तपाइँ गाउँको गतिविधिमा पूर्ण रूपमा संलग्न हुनुहुन्छ?

तपाईलाई रोग बारे के थाहा छ?

तपाईं कसरी उपचार प्राप्त गर्नुहुन्छ? के तपाइँ त्यसो गर्दा कुनै कठिनाइहरूको सामना गर्नुहुन्छ? के तपाइँ तपाइँको औषधि किन्नु हुन्छ ?

के तपाईले आफ्नो रोगको कारणले आर्थिक समस्याको सामना गर्नुपरेको छ?

तपाईं कुनै अन्य मुद्दाहरूमा छलफल गर्न चाहनुहुन्छ?
